# Dynamic imaging of Ostwald ripening in copper oxide nanoparticles by atomic resolution transmission Electron microscope

**DOI:** 10.1186/s42649-019-0019-z

**Published:** 2019-12-16

**Authors:** Na Yeon Kim

**Affiliations:** Center for Multidimensional Carbon Materials (CMCM), Institute for Basic Science (IBS), Ulsan, 44919 Republic of Korea

**Keywords:** Ostwald ripening, Atomic diffusion, Transmission Electron microscopy, Copper oxide nanoparticle, Atomic resolution image

## Abstract

Structural evolution of copper oxide nanoparticles is examined, especially with respect to Ostwald ripening under electron beam irradiation. Dissolution of the smaller particles into the larger one was clearly observed at the atomic scale using advanced transmission electron microscope.

## Description

**Fig. 1:**
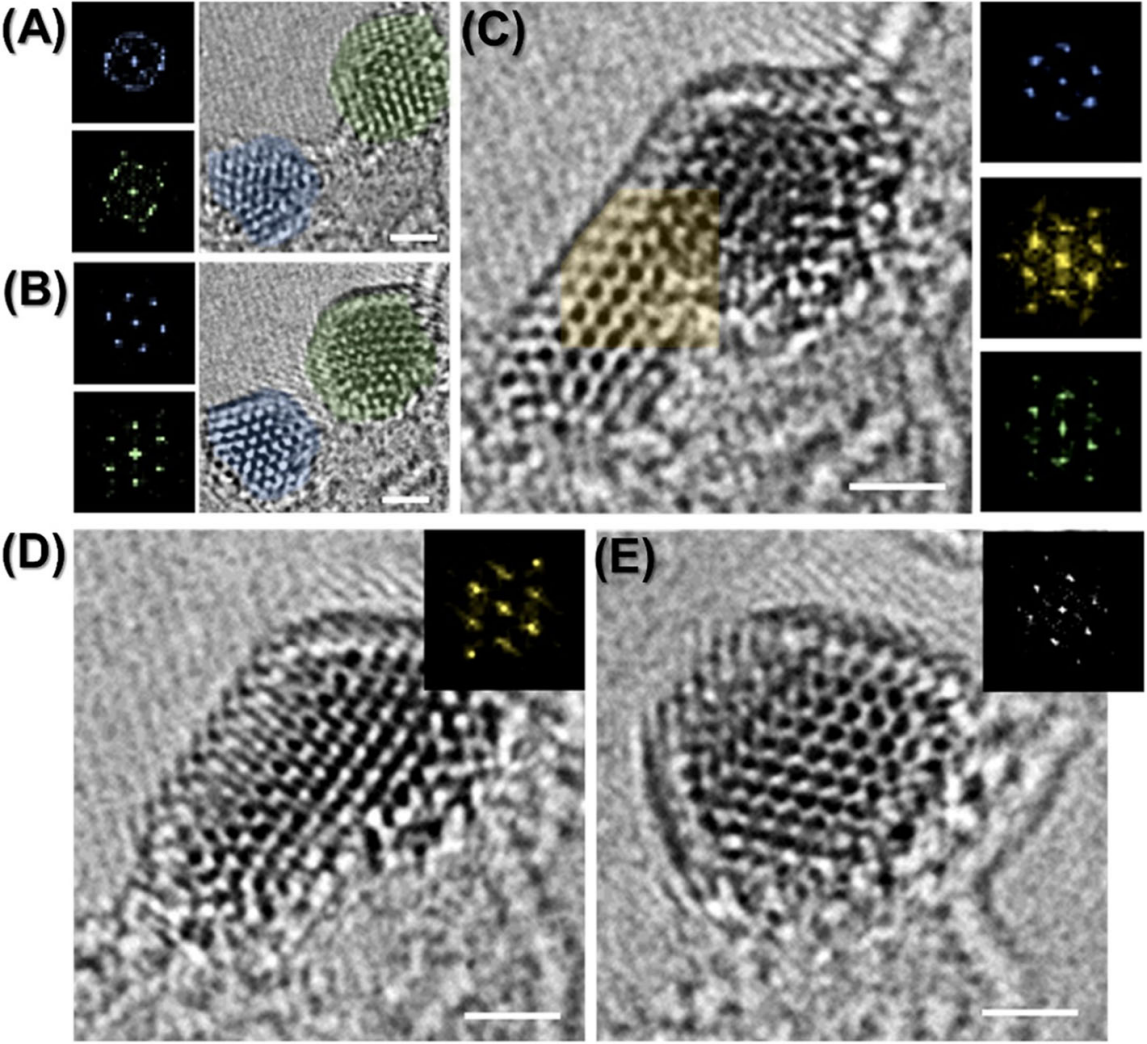
Atomic resolution TEM images with respect to Ostwald ripening in Cu_x_O NPs on graphene. All scale bars are 1 nm. **a**, **b** Crystallization in Cu_x_O NPs from the amorphous phase during electron beam irradiation. The gap of acquisition time between **a** to **b** is about 6 s. **b**-**d** Ostwald ripening process between two Cu_x_O NPs taken by 0.2 s time-series acquisition. **e** Crystallized Cu_x_O NP with hexagonal lattice structures 10 s after taking **d**

Ostwald ripening is a well-known crystal growth phenomenon, arising from unbalanced atomic diffusion from smaller nanoparticles with higher surface energy to bigger one (Voorhees 1985; Kuo et al. 2013; Ouyang et al. 2013). Transmission electron microscopy (TEM) is adequate to demonstrate the phenomenon associated with atomic diffusion and migration using state-of-the-art techniques (Bell et al. 2010). Herein, we show in-situ structural evolution in copper oxide nanoparticles (Cu_x_O NPs), especially in terms of crystallization and Ostwald ripening under electron beam irradiation in TEM. The amorphous copper oxide, widespread residues on CVD graphene surfaces (Lupina et al. 2015), shows crystallization from non-crystalline to hexagonal-like lattice structures with random orientation during electron-beam irradiation (Fig. 1 a). When the two crystallized blue and green Cu_x_O NPs become close to each other, Ostwald ripening occurs by atomic diffusion from the blue Cu_x_O NP to the green one. In particular, it appears that the smaller blue Cu_x_O NP with 2 nm in diameter rotated around 23 degree to diffuse into the larger green one just after 0.2 s acquisition time (Fig. 1b, c). On the other hand, the green Cu_x_O NP just rotated a fraction of degree. Misorientation in the growing Cu_x_O NP involves double reflections and blur in digital diffractograms (yellow insets of Fig. 1 c and d) and elongation on the outermost lattices (Fig. 1 d). After prolonged e-beam irradiation of approximately 10 s, it appears that the Cu_x_O NP was transformed into the well-crystallized structure of hexagonal lattices in absence of defects like twin boundaries (Fig. 1 e).

This image provides direct evidence of Ostwald ripening regarding the phenomenon that the smaller particles have higher solubility owing to higher surface energy by showing atomic dissolution and rotation of Cu_x_O NPs under atomic scale investigation.

## Data Availability

Not applicable. “Please contact author for data requests.”
